# Orthodontic patients’ knowledge, attitude, and practice regarding periodontal health

**DOI:** 10.1186/s12903-026-08750-4

**Published:** 2026-06-03

**Authors:** Jing Sun, Liang Chen, Lande Xue, Man Chen, Peipei Zhang, Hui Li, Shengnan Zhang, Yugang Sun

**Affiliations:** 1 Central Laboratory, Jinan Key Medical and Health Laboratory of Oral Diseases and Tissue Regeneration, Jinan Key Laboratory of Oral Diseases and Tissue Regeneration, Shandong Provincial Key Medical and Health Laboratory of Oral Diseases and Tissue Regeneration, Jinan Stomatological Hospital, Jinan, Shandong Province 250001 China; 2 Jinan Stomatological Hospital Shungeng Branch, Jinan Key Medical and Health Laboratory of Oral Diseases and Tissue Regeneration, Jinan Key Laboratory of Oral Diseases and Tissue Regeneration, Shandong Provincial Key Medical and Health Laboratory of Oral Diseases and Tissue Regeneration, Shandong Provincial Key Medical and Health Discipline of Oral Medicine, Jinan Stomatological Hospital, Jinan, Shandong Province 250001 China; 3 Jinan Stomatological Hospital East Branch, Central Laboratory, Jinan Key Medical and Health Laboratory of Oral Diseases and Tissue Regeneration, Jinan Key Laboratory of Oral Diseases and Tissue Regeneration, Shandong Provincial Key Medical and Health Laboratory of Oral Diseases and Tissue Regeneration, Jinan Stomatological Hospital, Jinan, Shandong Province 250001 China; 4 Hospital Infection Management Office, Central Laboratory, Jinan Key Medical and Health Laboratory of Oral Diseases and Tissue Regeneration, Jinan Key Laboratory of Oral Diseases and Tissue Regeneration, Shandong Provincial Key Medical and Health Laboratory of Oral Diseases and Tissue Regeneration, Jinan Stomatological Hospital, Jinan, Shandong Province 250001 China; 5 Department of Neurology, The People’s Hospital of Gaoqing District, Gaoqing, Shandong 256300 China

**Keywords:** Orthodontics, Periodontal Diseases, Oral Hygiene, Health Knowledge, Attitudes, Practice, Cross-Sectional Studies, Patient Education

## Abstract

**Background:**

This study aimed to assess the knowledge, attitudes, and practices (KAP) of orthodontic patients regarding periodontal health.

**Methods:**

A multicenter cross-sectional survey was conducted between December 2024 and May 2025 at Jinan Stomatological Hospital. Structured questionnaires were administered to collect demographic information and to evaluate participants’ KAP scores related to periodontal health. A cutoff value of 70% of the maximum possible score was applied to define adequate knowledge, positive attitude, and proactive practice.

**Results:**

A total of 2,638 valid questionnaires were included in the final analysis. Among the respondents, 1,636 (62.02%) were female, with a mean age of 31.59 ± 10.44 years. A total of 1,853 participants (70.24%) were undergoing orthodontic treatment for the first time. The mean scores for KAP were 12.54 ± 3.94 (possible range: 0–18), 46.00 ± 4.13 (possible range: 10–50), and 39.30 ± 5.01 (possible range: 9–45), respectively. Structural equation modeling (SEM) demonstrated that knowledge was positively associated with attitude (β = 0.493, *P* < 0.001) and practice (β = 0.753, *P* < 0.001), and that attitude was positively associated with practice (β = 1.087, *P* < 0.001). In addition, knowledge showed an indirect association with practice mediated by attitude (β = 0.535, *P* < 0.001).

**Conclusion:**

Orthodontic patients exhibited insufficient knowledge but maintained generally positive attitudes and proactive practices regarding periodontal health. These findings highlight the importance of understanding periodontal health literacy among orthodontic patients within a descriptive KAP framework.

**Clinical trial number:**

Not applicable.

**Supplementary Information:**

The online version contains supplementary material available at 10.1186/s12903-026-08750-4.

## Background

Periodontal disease represents one of the most significant oral health challenges worldwide and is a leading cause of tooth loss in adults, posing particular risks during orthodontic treatment [1]. Its management becomes more complex during orthodontic therapy, as mechanical factors introduced by orthodontic appliances complicate oral hygiene and may exacerbate periodontal conditions [2]. Clear aligners have been reported to provide more favorable periodontal outcomes than fixed appliances, with significantly lower plaque index, gingival index, and probing depth values [3]. Concurrently, the demand for orthodontic treatment has increased substantially, particularly in China, where treatment rates among contemporary college freshmen exceed 48.64%, representing a marked rise compared with previous decades [4]. This rapid expansion in orthodontic care, together with the inherent periodontal risks associated with orthodontic appliances, underscores the importance of maintaining optimal periodontal health throughout treatment.

During orthodontic treatment, patients encounter unique challenges in preserving periodontal health, as orthodontic appliances facilitate microbial adhesion by creating additional surfaces and retentive areas that disrupt the natural oral ecological balance [5]. Individuals undergoing orthodontic treatment exhibit both qualitative and quantitative alterations in oral microbiota activity compared with untreated individuals. Increased retention of supra- and subgingival plaque during treatment may result in adverse outcomes, including gingivitis and white spot lesions [6]. Within the first three months of orthodontic treatment, the prevalence of gingivitis has been reported to increase from 18.3% to 52.5%, indicating a rapid decline in periodontal status [7]. This elevated risk emphasizes the necessity of evaluating and enhancing orthodontic patients’ knowledge, attitudes, and practices related to periodontal health maintenance.

The Knowledge, Attitude, and Practice (KAP) framework is a widely used diagnostic tool in health literacy research, based on the premise that knowledge influences attitudes, which subsequently shape health-related behaviors [8]. Existing studies have revealed notable deficiencies in periodontal health literacy among orthodontic patients. For example, one study reported that only 24 participants (8%) correctly answered knowledge-related questions [9]. Adult orthodontic patients have also been shown to hold negative attitudes toward fixed orthodontic treatment with respect to periodontal health, with longer treatment duration adversely affecting attitudes [9]. Even among adults with advanced periodontitis seeking orthodontic treatment, more than half express interest in orthodontic care, although their knowledge of periodontal disease and oral health status significantly influences this interest [10]. Nevertheless, comprehensive KAP studies focusing on periodontal health among Chinese orthodontic patients remain limited, despite the rapid growth of orthodontic treatment in China and the potential influence of cultural factors on health behaviors and perceptions.

This study aimed to assess the knowledge, attitudes, and practices regarding periodontal health among orthodontic patients in China.

## Methods

### Study design and participants

This multicenter cross-sectional study was conducted between December 2024 and May 2025 at Jinan Stomatological Hospital and included individuals undergoing orthodontic treatment. Ethical approval was obtained from the Ethics Committee of Jinan Stomatological Hospital (Approval No.: JNSKQYY-2024-023). Prior to participation, all respondents received detailed information regarding the study objectives, voluntary participation, and data confidentiality, and electronic informed consent was obtained through the online questionnaire system. Anonymity was ensured, as no personally identifiable information was collected, and all data were stored in password-protected files accessible only to the research team. All procedures were performed in accordance with the Declaration of Helsinki.

Participants were eligible for inclusion if they met all of the following criteria: (1) currently receiving orthodontic treatment; (2) aged 18 years or older; and (3) able to understand the study objectives and provide informed consent voluntarily. Exclusion criteria included a diagnosis of severe systemic diseases, such as advanced malignancies or significant cardiopulmonary disorders, or a history of periodontal surgical treatment.

### Questionnaire

The questionnaire was developed based on a review of relevant literature [8, 11, 12] and existing clinical guidelines [13]. An initial version was drafted and subsequently reviewed by one specialist in periodontics and one specialist in orthodontics. Revisions were made according to their recommendations to improve content validity, including the addition of items addressing clinical symptoms of periodontitis and information related to the type and duration of orthodontic appliance use. A pilot survey involving 72 participants was then conducted to evaluate the psychometric properties of the instrument. The results demonstrated good internal consistency, with a Cronbach’s α coefficient of 0.895 for the overall scale. The Cronbach’s α values for the KAP subscales were 0.860, 0.758, and 0.741, respectively. The relatively lower α values for the attitude and practice subscales may be attributable to the limited number of items and the multidimensional nature of these constructs. Nonetheless, all coefficients exceeded the recommended threshold of 0.70, indicating acceptable reliability.

The finalized questionnaire, administered in Chinese, comprised four main sections: demographic characteristics, including age, gender, education level, occupation, and monthly income; knowledge; attitude; and practice (Additional file. Questionnaire). The KAP sections were designed to comprehensively assess participants’ understanding, perceptions, and behaviors. The knowledge section consisted of nine items, each scored using a three-point scale: “Very familiar” (2 points), “Heard of it” (1 point), and “Unfamiliar” (0 points), resulting in a total possible score ranging from 0 to 18. The attitude section included ten items assessed using a five-point Likert scale from “Strongly agree” to “Strongly disagree,” with corresponding scores from 5 to 1 and a total score range of 10 to 50. The practice section also comprised nine items, evaluated using a five-point frequency scale from “Never” (1 point) to “Always” (5 points), yielding a total score range of 9 to 45. To facilitate interpretation, cutoff values were established to define adequate knowledge, positive attitudes, and proactive practices, with each dimension considered satisfactory when the score reached or exceeded 70% of the maximum possible score, in accordance with previous studies [8, 14].

### Questionnaire distribution and quality control

Data collection was conducted electronically using an online questionnaire platform. A convenience sampling method was adopted, whereby quick response (QR) codes linking to the questionnaire were made available in the orthodontic departments of the main hospital and six affiliated branches of Jinan Stomatological Hospital. Patients attending these clinics were invited to participate by scanning the QR code. A trained team of ten research assistants facilitated the distribution process, provided clarification when necessary, and ensured that responses were completed independently and without external influence.

To enhance data reliability and ensure analytical integrity, predefined criteria were applied to identify and exclude invalid or biased responses. Questionnaires completed in less than 90 s were excluded, as such short completion times were considered indicative of inadequate engagement. Responses containing implausible values, such as ages outside a biologically reasonable range, were removed to prevent distortion of statistical analyses. In addition, questionnaires exhibiting uniform response patterns across all items were excluded, as these suggested inattentive or insincere participation. Logical inconsistencies within responses, such as selecting mutually contradictory options within the same question, also led to exclusion. For example, participants who simultaneously selected “no symptoms” and one or more specific symptoms in the comorbidity section were removed. Finally, questionnaires containing missing data were excluded to maintain consistency in the statistical modeling.

### Statistical analysis

Statistical analyses were performed using STATA version 17.0 (StataCorp LLC, College Station, TX, USA). Continuous variables were presented as means ± standard deviations (SD), and normality was assessed using the Kolmogorov–Smirnov test. Variables following a normal distribution were analyzed using Student’s t test or one-way analysis of variance (ANOVA), whereas non-normally distributed variables were analyzed using the Wilcoxon–Mann–Whitney U test or the Kruskal–Wallis test. Categorical variables were expressed as frequencies and percentages [n (%)]. Spearman’s rank correlation coefficient was used to evaluate associations among KAP scores. To further examine the conceptual relationships among knowledge, attitude, practice, and general information, structural equation modeling (SEM) was conducted in accordance with the KAP theoretical framework. The model was applied in an exploratory and predictive manner to assess potential pathways consistent with the framework, including whether attitude mediated the association between knowledge and practice. Both direct and indirect effects were estimated and compared. Model fit was evaluated using the root mean square error of approximation (RMSEA), standardized root mean square residual (SRMR), Tucker–Lewis index (TLI), and comparative fit index (CFI). Acceptable model fit was defined as RMSEA < 0.08, SRMR < 0.08, TLI > 0.80, and CFI > 0.80. A two-sided P value of less than 0.05 was considered statistically significant.

## Results

A total of 3,460 participants were initially recruited. Of these, 39 were excluded due to response times of less than 90 s, 1 due to an abnormal age value, 777 due to age below 18 years, 1 due to logical inconsistency, and 4 due to missing data. Ultimately, 2,638 valid questionnaires were included in the final analysis, yielding a valid response rate of 76.26%.

### Demographic characteristics and KAP scores

Among the participants, 1,636 (62.02%) were female, with a mean age of 31.59 ± 10.44 years. A total of 1,052 participants (39.88%) held a bachelor’s degree, and 1,853 (70.24%) were wearing an orthodontic appliance for the first time. Additionally, 911 participants (34.53%) had been undergoing orthodontic treatment for 1 to 2 years, while 368 (13.95%) had received treatment for less than 3 months. Fixed appliances were the most commonly used treatment modality (68.88%), followed by clear aligners (21.80%) and removable appliances (9.33%). Furthermore, 67.06% of participants reported having family members who had previously undergone orthodontic treatment **(**Table 1**)**.

**Table 1:** Demographic characteristics and KAP scores

*N* = 2638	*N* (%)	Knowledge score		Attitude score		Practice score	
		Mean ± SD	*P*	Mean ± SD	*P*	Mean ± SD	*P*
Total score		12.54 ± 3.94		46.00 ± 4.13		39.30 ± 5.01	
Gender			< 0.001		< 0.001		< 0.001
Male	1002(37.98)	15.61 ± 2.49		49.04 ± 1.93		43.11 ± 2.62	
Female	1636(62.02)	10.65 ± 3.44		44.12 ± 4.00		36.96 ± 4.68	
Age (years old)	31.59 ± 10.44						
Residence			< 0.001		< 0.001		< 0.001
Rural	201(7.62)	16.29 ± 2.12		49.36 ± 1.85		43.84 ± 2.16	
Urban	2437(92.38)	12.22 ± 3.89		45.72 ± 4.14		38.92 ± 4.99	
Education			< 0.001		< 0.001		< 0.001
Junior high school or below	242(9.17)	16.21 ± 1.71		49.57 ± 0.88		43.82 ± 1.42	
Senior high school / Vocational high school	492(18.65)	15.60 ± 2.42		49.03 ± 1.80		43.16 ± 2.42	
Junior college	551(20.89)	14.28 ± 3.20		47.78 ± 2.92		41.48 ± 3.77	
Bachelor’s degree	1052(39.88)	10.52 ± 3.27		44.11 ± 3.90		36.83 ± 4.56	
Master’s degree or above	301(11.41)	8.408 ± 2.24		41.47 ± 3.23		34.00 ± 3.35	
Monthly income per capita			< 0.001		< 0.001		< 0.001
< 2000	65(2.46)	16.12 ± 2.56		49.67 ± 1.30		44.01 ± 1.85	
2000–5000	218(8.26)	16.22 ± 2.23		49.46 ± 1.67		43.72 ± 2.26	
5000–10,000	849(32.18)	15.05 ± 2.68		48.55 ± 2.25		42.52 ± 2.95	
10,000–20,000	425(16.11)	12.44 ± 3.46		46.25 ± 3.53		39.55 ± 4.26	
> 20,000	339(12.85)	11.13 ± 3.28		44.66 ± 3.97		37.41 ± 4.59	
Prefer not to disclose	742(28.13)	8.943 ± 2.64		42.19 ± 3.47		34.61 ± 3.79	
Marital status			< 0.001		< 0.001		< 0.001
Unmarried	1639(62.13)	14.43 ± 3.23		48.00 ± 2.96		41.76 ± 3.73	
Married	967(36.66)	9.464 ± 2.86		42.77 ± 3.64		35.34 ± 4.15	
Other	32(1.21)	8 ± 2.48		40.84 ± 3.25		32.75 ± 3.52	
First time wearing an orthodontic appliance			< 0.001		< 0.001		< 0.001
Yes	1853(70.24)	14.02 ± 3.42		47.56 ± 3.31		41.22 ± 4.16	
No, have been wearing one for a while	785(29.76)	9.022 ± 2.63		42.29 ± 3.45		34.76 ± 3.78	
Type of orthodontic treatment			< 0.001		< 0.001		< 0.001
Fixed appliance (bracket-type orthodontic appliance)	1817(68.88)	14.05 ± 3.42		47.63 ± 3.25		41.34 ± 4.02	
Bracket-free clear aligners (invisible braces)	575(21.8)	9.504 ± 2.84		42.78 ± 3.55		35.26 ± 3.97	
Removable appliance (braces that can be taken off by oneself)	246(9.33)	8.369 ± 2.27		41.39 ± 3.19		33.63 ± 3.47	
Duration of wearing orthodontic appliance			< 0.001		< 0.001		< 0.001
Less than 3 months	368(13.95)	16.20 ± 2.12		49.41 ± 1.61		43.68 ± 2.29	
3–6 months	361(13.68)	15.35 ± 2.57		48.91 ± 1.92		42.99 ± 2.38	
6–12 months	589(22.33)	14.25 ± 3.08		47.82 ± 2.88		41.50 ± 3.60	
1–2 years	911(34.53)	10.56 ± 3.25		44.12 ± 3.93		36.95 ± 4.56	
Over 2 years	409(15.5)	8.657 ± 2.47		41.89 ± 3.25		34.17 ± 3.52	
Family members who have undergone orthodontic treatment			< 0.001		< 0.001		< 0.001
Yes	1769(67.06)	14.27 ± 3.23		47.85 ± 2.99		41.48 ± 3.98	
No	869(32.94)	9.004 ± 2.69		42.21 ± 3.48		34.87 ± 3.83	

**Fig. 1:**
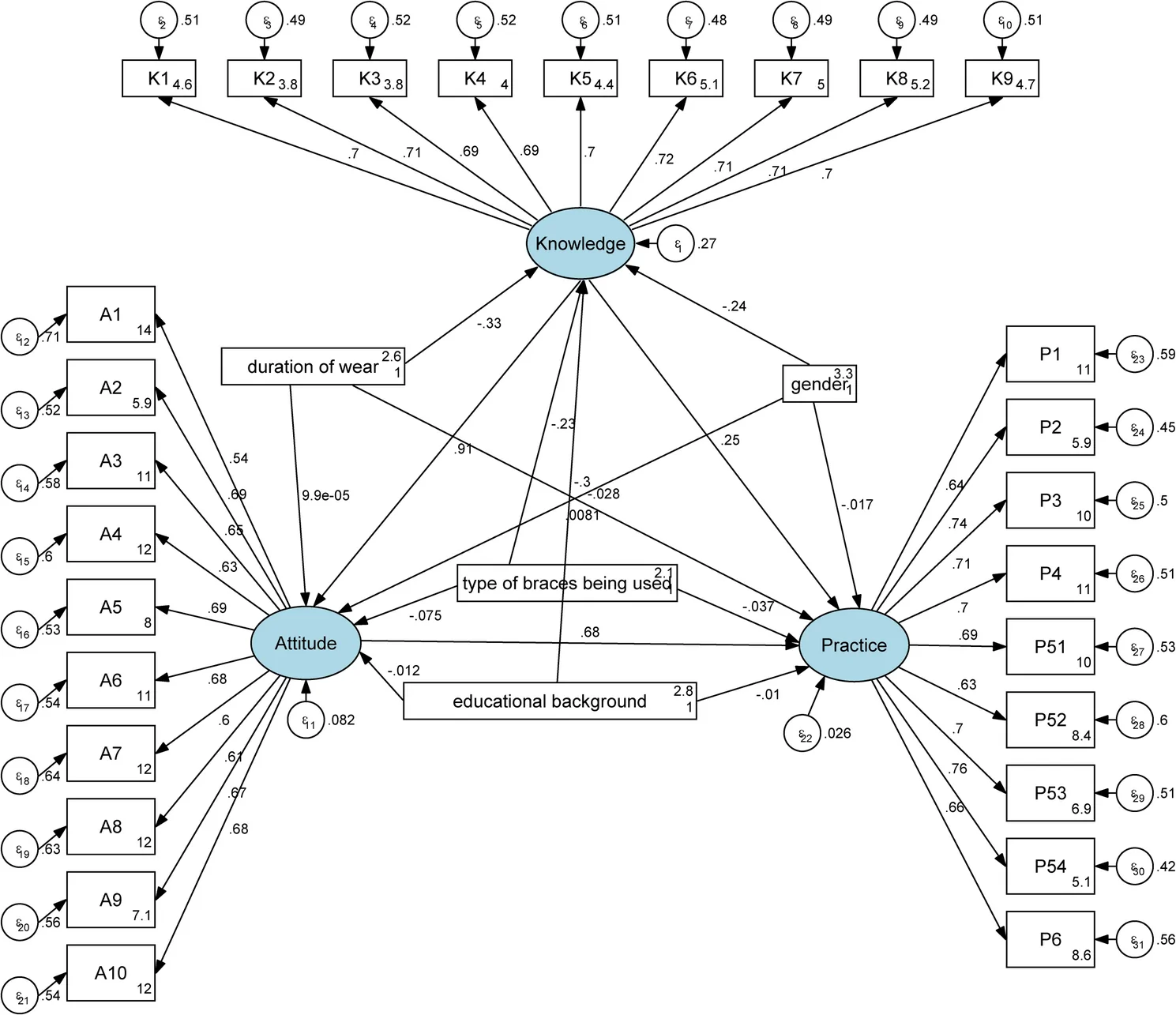
SEM analysis. Ellipses represent latent variables (unobserved constructs), including overall knowledge, attitude, and practice. Rectangles represent observed variables (measured questionnaire items) that serve as indicators of the latent variables. Circles represent error terms or residuals associated with the observed variables. Single-headed arrows indicate hypothesized directional relationships (pathways) between latent variables and from latent variables to observed variables, with standardized path coefficients labeled on the arrows

Regarding oral health history and periodontal symptoms, more than half of the participants (52.08%) reported a history of dental caries, and 46.17% reported gingival bleeding. Gingival swelling (38.17%) and gingival pain (38.48%) were also frequently reported, whereas 20.02% of participants reported experiencing tooth mobility. Only a small proportion of respondents (0.27%) reported previous tooth loss, and 2.69% reported no dental or gingival problems. Most participants (91.32%) reported obtaining periodontal health information from dentists, followed by new media sources (48.33%) and communication with family or friends (44.31%) (FigureS1A, B).

The mean KAP scores were 12.54 ± 3.94 (range: 0 to 18), 46.00 ± 4.13 (range: 10 to 50), and 39.30 ± 5.01 (range: 9 to 45), respectively. Analysis of demographic characteristics demonstrated that KAP scores differed significantly according to gender, residence, education level, monthly per capita income, marital status, orthodontic appliance uses and duration, type of orthodontic treatment, and whether family members had undergone orthodontic treatment (all *P* < 0.001). Notably, knowledge scores showed an inverse association with education level. Participants with a junior high school education or below achieved the highest mean knowledge score (16.21 ± 1.71), whereas those with a master’s degree or above had the lowest mean score (8.41 ± 2.24) (Table 1).

### Distribution of KAP responses

Analysis of the distribution of knowledge items revealed that the three statements with the lowest proportions of participants selecting “Very familiar” were “Bleeding gums may be an early symptom of periodontal disease” (K3), selected by 31.46% of participants; “Orthodontic treatment may increase the risk of periodontal problems such as gingival inflammation” (K2), selected by 33.66%; and “Periodontal disease may lead to loose teeth or even tooth loss” (K4), selected by 36.09% (Table 2).

**Table 2:** Distribution of knowledge dimension responses

Knowledge items, *n* (%)	Very familiar	Heard of it	Unclear
1. Periodontitis is caused by a variety of factors including poor oral hygiene, smoking, and genetic predisposition.	1233(46.74)	1304(49.43)	101(3.83)
2. Orthodontic treatment may increase the risk of periodontal problems such as gingival inflammation.	888(33.66)	1292(48.98)	458(17.36)
3. Bleeding gums may be an early symptom of periodontal disease.	830(31.46)	1426(54.06)	382(14.48)
4. Periodontal disease may lead to loose teeth or even tooth loss.	952(36.09)	1398(52.99)	288(10.92)
5. Periodontal disease can be prevented through regular cleaning and proper care.	1143(43.33)	1351(51.21)	144(5.46)
6. Proper brushing techniques can effectively prevent periodontal diseases.	1472(55.8)	1134(42.99)	32(1.21)
7. Using dental floss helps clean interdental spaces and reduces gingivitis.	1486(56.33)	1087(41.21)	65(2.46)
8. Regular dental check-ups help with early detection of periodontal problems.	1586(60.12)	1023(38.78)	29(1.1)
9. Periodontal disease not only affects oral health but may also impact overall health.	1345(50.99)	1186(44.96)	107(4.06)

Responses in the attitude dimension indicated that 30.21% of participants selected a neutral response regarding whether periodontal health affects the effectiveness of orthodontic treatment (A2). In addition, 9.74% expressed neutrality concerning whether good oral hygiene habits can prevent most periodontal problems (A6). Furthermore, 5.23% of participants reported unwillingness to learn more about periodontal health during orthodontic treatment (A10) (Table 3).

**Table 3:** Distribution of attitude dimension responses

Attitude items, *n* (%)	Strongly agree	Agree	Neutral	Disagree	Strongly disagree
1. I believe maintaining good periodontal health is important during orthodontic treatment.	2151(81.54)	478(18.12)	9(0.34)	/	/
2. I believe periodontal health affects the effectiveness of orthodontic treatment.	1643(62.28)	70(2.65)	797(30.21)	87(3.3)	41(1.55)
3. I believe patients should have regular periodontal check-ups after orthodontic treatment.	1855(70.32)	754(28.58)	23(0.87)	6(0.23)	/
4. I believe orthodontic patients should receive professional guidance on periodontal health.	1925(72.97)	693(26.27)	20(0.76)	/	/
5. I am concerned about periodontal health issues occurring during orthodontic treatment.	1925(72.97)	693(26.27)	20(0.76)	/	/
6. I believe that good oral hygiene habits can prevent most periodontal problems.	1343(50.91)	992(37.6)	257(9.74)	46(1.74)	/
7. I trust my dentist’s professional competence regarding periodontal health.	1772(67.17)	827(31.35)	39(1.48)	/	/
8. I am willing to follow the dentist’s instructions for daily oral cleaning and care.	1995(75.63)	622(23.58)	21(0.8)	/	/
9. Orthodontic treatment has made maintaining my oral hygiene more difficult.	1956(74.15)	657(24.91)	20(0.76)	5(0.19)	/
10. I am willing to learn more about periodontal health during orthodontic treatment.	1440(54.59)	891(33.78)	161(6.1)	138(5.23)	8(0.3)

Responses in the practice dimension showed that 17.48% of participants rarely and 5.69% never used mouthwash to maintain oral hygiene (P5.4). Moreover, 11.52% rarely and 0.95% never visited a periodontal clinic for professional periodontal cleaning (P2), while 4.80% rarely and 0.38% never used dental floss or other auxiliary tools to clean interdental spaces (P5.3) (Table 4).

**Table 4:** Distribution of practice dimension responses

Practice items, *n* (%)	Always	Often	Sometimes	Rarely	Never
1. I attend orthodontic follow-up appointments at the hospital as scheduled by my doctor.	1878(71.19)	675(25.59)	84(3.18)	/	1(0.04)
2. I regularly visit the periodontal clinic for professional periodontal cleaning.	1003(38.02)	831(31.5)	475(18.01)	304(11.52)	25(0.95)
3. During orthodontic treatment, I follow the doctor’s advice for daily oral care.	1587(60.16)	953(36.13)	97(3.68)	1(0.04)	/
4. I follow the doctor’s instructions regarding daily cleaning of orthodontic appliances.	1644(62.32)	940(35.63)	53(2.01)	1(0.04)	/
5. In daily life, I pay attention to the following:					
5.1 Using the correct brushing technique	1675(63.5)	908(34.42)	37(1.4)	18(0.68)	/
5.2 Brushing my teeth at least twice a day	1754(66.49)	650(24.64)	191(7.24)	43(1.63)	/
5.3 Using dental floss or other tools to clean between teeth	1234(46.78)	945(35.82)	322(12.21)	127(4.81)	10(0.38)
5.4 Using mouthwash to maintain oral hygiene	822(31.16)	568(21.53)	637(24.15)	461(17.48)	150(5.69)
6. When I experience oral discomfort, I promptly go to the hospital and seek advice from healthcare professionals.	1755(66.53)	633(24)	226(8.57)	24(0.91)	/

### Correlations between KAP

Correlation analysis demonstrated that knowledge was positively correlated with attitude (*r* = 0.864, 95% CI: 0.854–0.874, *P* < 0.001) and practice (*r* = 0.873, 95% CI: 0.863–0.882, *P* < 0.001). Attitude was also positively correlated with practice (*r* = 0.872, 95% CI: 0.863–0.881, *P* < 0.001) (Table **S1**).

### Interactions between KAP

The structural equation model demonstrated good fit to the data, with fit indices exceeding the predefined threshold values (RMSEA = 0.018; SRMR = 0.014; TLI = 0.990; CFI = 0.989), indicating satisfactory model performance (Table **S2**). SEM results showed that knowledge was positively associated with attitude (β = 0.493, *P* < 0.001) and practice (β = 0.753, *P* < 0.001), and attitude was also positively associated with practice (β = 1.087, *P* < 0.001). In addition, a significant indirect association between knowledge and practice mediated by attitude was observed (β = 0.535, *P* < 0.001) (Table 5 and Fig. 1). Furthermore, type of orthodontic appliance, duration of appliance wear, gender, and educational background demonstrated statistically significant total effects on KAP (all *P* < 0.001). Their effects on attitude and practice were partially mediated through knowledge or attitude, with indirect effects remaining statistically significant (all *P* < 0.001) **(**Table 5**)**.

**Table 5:** SEM direct and indirect effects

Model paths		Total effects		Direct Effect		Indirect effect	
		β(95%CI)	*P*	β(95%CI)	*P*	β(95%CI)	*P*
Knowledge							
	Type of braces being used	-0.141 (-0.158, -0.124)	< 0.001	-0.141 (-0.158, -0.124)	< 0.001		
	Duration of wear	-0.102 (-0.112, -0.092)	< 0.001	-0.102 (-0.112, -0.092)	< 0.001		
	Gender	-0.198 (-0.223, -0.173)	< 0.001	-0.198 (-0.223, -0.173)	< 0.001		
	Educational background	-0.103 (-0.114, -0.092)	< 0.001	-0.103 (-0.114, -0.092)	< 0.001		
Attitude							
	Knowledge	0.493 (0.448, 0.537)	< 0.001	0.493 (0.448, 0.537)	< 0.001		
	Type of braces being used	-0.094 (-0.105, -0.083)	< 0.001	-0.025 (-0.034, -0.015)	< 0.001	-0.069 (-0.079, -0.059)	< 0.001
	Duration of wear	-0.050 (-0.056, -0.044)	< 0.001	0.000 (-0.005, 0.005)	0.995	-0.050 (-0.056, -0.044)	< 0.001
	Gender	-0.094 (-0.109, -0.079)	< 0.001	0.004 (-0.009, 0.016)	0.586	-0.097 (-0.112, -0.083)	< 0.001
	Educational background	-0.053 (-0.060, -0.046)	< 0.001	-0.002 (-0.008, 0.003)	0.434	-0.051 (-0.057, -0.044)	< 0.001
Practice							
	Knowledge	0.753 (0.693, 0.814)	< 0.001	0.218 (0.104, 0.332)	< 0.001	0.535 (0.427, 0.644)	< 0.001
	Attitude	1.087 (0.868, 1.306)	< 0.001	1.087 (0.868, 1.306)	< 0.001		
	Type of braces being used	-0.152 (-0.168, -0.136)	< 0.001	-0.019 (-0.032, -0.007)	0.002	-0.133 (-0.149, -0.117)	< 0.001
	Duration of wear	-0.084 (-0.093, -0.075)	< 0.001	-0.008 (-0.014, -0.001)	0.034	-0.077 (-0.086, -0.068)	< 0.001
	Gender	-0.157 (-0.179, -0.135)	< 0.001	-0.012 (-0.029, 0.005)	0.167	-0.145 (-0.166, -0.124)	< 0.001
	Educational background	-0.083 (-0.093, -0.074)	< 0.001	-0.003 (-0.011, 0.004)	0.422	-0.080 (-0.090, -0.071)	< 0.001

## Discussion

Despite generally favorable attitudes and consistent engagement in oral hygiene practices, orthodontic patients exhibited insufficient knowledge regarding periodontal health. These findings suggest that the incorporation of structured periodontal education into orthodontic care may enhance foundational knowledge and reinforce long-term behavioral adherence.

This study assessed KAP related to periodontal health among orthodontic patients using a structured KAP framework. The results revealed a clear imbalance among the three dimensions. Although most participants demonstrated positive attitudes and reported adherence to recommended oral health behaviors, their level of periodontal health knowledge was comparatively inadequate. Similar patterns have been reported in previous studies conducted in both orthodontic and periodontal settings. For example, a recent cross-sectional study of orthodontic patients found that, despite generally positive attitudes and appropriate oral health behaviors, knowledge related to periodontal health remained insufficient [15]. Likewise, another study reported that orthodontic patients frequently exhibited limited understanding of periodontal health, particularly regarding the causes and consequences of periodontitis, even though they expressed willingness to comply with oral hygiene instructions [9]. Additionally, research involving adults with severe periodontal disease indicated strong interest in orthodontic treatment but inadequate awareness of the associated risks [10]. Collectively, these findings suggest that knowledge gaps are common in orthodontic populations, particularly when preventive information is not consistently emphasized during clinical encounters. Social desirability bias may also contribute, as participants may overreport favorable attitudes or behaviors to conform to perceived expectations in self-reported surveys. Furthermore, adherence to oral hygiene practices may be influenced by instructions from clinicians or caregivers, even when underlying knowledge remains limited.

The present study further demonstrated that knowledge exerted both direct and indirect effects on practice, with attitude serving as a significant mediating factor, as confirmed by SEM analysis. This pattern is consistent with the theoretical assumptions of the KAP framework, although causal relationships cannot be established due to the cross-sectional study design [8]. In the current analysis, knowledge positively influenced attitudes (β = 0.51) and directly affected practices (β = 1.12), while also indirectly influencing practices through attitudes (β = 0.58). Comparable mechanisms have been reported in other areas of health behavior research [16, 17]. Nevertheless, a substantial proportion of participants remained uncertain about whether periodontal health influences orthodontic treatment outcomes. This finding indicates that even when factual knowledge is present, insufficient perceived relevance may reduce motivation to adopt preventive behaviors. Therefore, periodontal education within orthodontic care should not only provide factual information but also emphasize its personal relevance and clinical importance.

Demographic comparisons further identified groups that may benefit from targeted interventions. SEM analysis revealed that orthodontic appliance type, duration of appliance wear, gender, and educational background exerted significant total effects on KAP, indicating that these baseline characteristics systematically influenced periodontal health-related behaviors. Specifically, participants using clear aligners or removable appliances exhibited lower knowledge and attitude scores than those treated with fixed appliances, with these effects partially mediated through reduced knowledge levels. This pattern may reflect the perception that aligners and removable appliances are easier to manage and pose fewer periodontal risks, leading to decreased vigilance in oral hygiene behaviors [18]. In addition, longer treatment duration was consistently associated with lower KAP scores, with both direct and indirect effects on attitude and practice mediated by knowledge. Participants undergoing orthodontic treatment for more than one year demonstrated poorer behavioral performance, which may be attributable to treatment fatigue, reduced risk perception, or diminished responsiveness to repeated oral health education. Similar trends have been observed in previous studies, in which prolonged exposure to routine medical or dental interventions was associated with decreased receptivity to preventive health messages [19, 20]. Gender and educational background also significantly influenced KAP outcomes. In this study, male participants exhibited higher KAP scores than female participants, while educational level was inversely associated with KAP, with lower scores observed among individuals with higher educational attainment. Gender-related differences in oral health KAP have been widely reported, although the direction of these associations varies across populations [21], suggesting the influence of sociocultural and behavioral factors. Although higher education is generally associated with improved health literacy, the inverse relationship observed in this study may reflect greater critical self-evaluation or heightened awareness of inadequate practices among more highly educated individuals, a phenomenon previously reported in KAP research [22].

Within each KAP domain, the present findings identified specific educational content areas requiring reinforcement. These included early symptom recognition and awareness of the systemic effects of periodontal disease within the knowledge domain, understanding of the relationship between periodontal health and adverse consequences within the attitude domain, and adherence to adjunctive oral hygiene behaviors, such as dental floss use and professional periodontal cleaning, within the practice domain. Analysis of the knowledge domain revealed insufficient recognition of common periodontal symptoms, particularly gingival bleeding, with only 31.46% of participants correctly identifying it as an early sign of periodontal disease. Limited symptom awareness may delay timely care seeking and reduce the effectiveness of early intervention. Previous studies have demonstrated that individuals who are able to recognize early signs of periodontal disease are more likely to engage in preventive behaviors and achieve improved clinical outcomes [23, 24]. Interestingly, participants with lower levels of formal education achieved higher scores in knowledge and practice domains. This finding, which has also been reported in earlier studies, may be attributed to more frequent interactions with dental professionals during treatment, thereby compensating for limited health literacy through repeated verbal instruction [23, 24]. These results suggest that patients with lower educational attainment may benefit more from provider-led education than from written materials, and that this approach should be sustained. With respect to practice behaviors, although daily toothbrushing was widely reported, behaviors requiring greater effort, such as the use of dental floss or mouthwash, were less consistently performed. For example, 17.48% of participants reported rarely using mouthwash, and only 46.78% reported regular use of dental floss. This discrepancy has also been observed in previous research, in which more complex or time-consuming behaviors were less frequently adopted even among individuals with positive attitudes [25, 26]. Furthermore, participants undergoing long-term orthodontic treatment were less likely to maintain these practices, possibly due to declining motivation or competing priorities.

Given that more than 90% of participants identified dentists as their primary source of periodontal health information, chairside education remains a critical opportunity for intervention. However, orthodontic appointments are often time constrained, making sustained educational messaging challenging. To address this limitation, brief educational modules delivered digitally within clinical settings or via mobile platforms may help reinforce essential information. In addition, nearly half of the participants reported obtaining oral health information through social media platforms such as WeChat or Xiaohongshu. These platforms may serve as effective adjuncts for continuous education, particularly through reminder systems, short educational videos, or behavior-tracking applications [27, 28]. Future educational initiatives should capitalize on patients’ preferences for digital media to ensure that periodontal health education remains accessible and engaging throughout orthodontic treatment.

Several limitations of this study should be acknowledged. First, although this was a multicenter investigation, all participating centers were located within the same geographic region, which may limit the generalizability of the findings to other regions or countries. Differences in population characteristics, clinical environments, and cultural contexts may influence KAP patterns, and caution is therefore warranted when extrapolating these results. Second, the cross-sectional design precludes causal inference regarding the relationships among knowledge, attitudes, and practices. Although SEM provides insight into potential pathways, the observed associations remain theoretical rather than causal. Third, reliance on self-reported questionnaires may have introduced response bias, as participants could have overestimated their attitudes or behaviors related to periodontal care. Additionally, dental pathology data were not collected in this study. Social desirability bias may also have influenced responses, leading some participants to report more favorable oral hygiene behaviors or attitudes. Due to practical constraints and the need for consistency across study centers, standardized periodontal examinations were not feasible for all participants, and objective clinical indicators were therefore not included as measures of practice. Future studies should integrate self-reported data with objective clinical assessments to improve validity. Moreover, variations in sample characteristics, including the relatively young age distribution, uneven educational levels, predominance of fixed-appliance users, and heterogeneity among orthodontic appliance types, may have influenced the observed KAP differences and should be considered when interpreting the generalizability of the findings.

## Conclusions

In conclusion, orthodontic patients demonstrated insufficient knowledge but maintained generally positive attitudes and engaged in proactive practices regarding periodontal health. These findings highlight the importance of further investigation into how targeted educational interventions may support periodontal health throughout orthodontic treatment.

## Supplementary Information

Supplementary Material 1.

Supplementary Material 2.

Supplementary Material 3.

## Data Availability

All data generated or analyzed during this study are included in this published article.
