# Supplementary material for: Orthodontic patients’ knowledge, attitude, and practice regarding periodontal health
Source: BMC Oral Health. 2026 Jun 3;26:1569. doi: 10.1186/s12903-026-08750-4 (PMC13504998; doi:10.1186/s12903-026-08750-4)
Supplement: Supplementary file 1 [file 12903_2026_8750_MOESM1_ESM.docx]

Questionnaire ID:

Dear Participant,

We are medical researchers from the hospital and sincerely invite you to participate in our research project. This study aims to understand the knowledge, attitudes, and practices of orthodontic patients regarding periodontal health, providing a basis for developing scientific intervention strategies. These strategies may help more people improve their health in the future. Your participation in this study is voluntary. The study has been approved by the Ethics Review Committee. If you agree to participate, please read the following instructions.

1. Please complete the questionnaire. There are no right or wrong answers; simply respond based on your actual situation. If you encounter any problems during the process, feel free to consult us. Once completed, please submit the questionnaire promptly.

2. This is a simple survey and will not cause any harm to your physical or psychological health. However, it may involve some personal information, such as your gender and age. We will strictly maintain confidentiality and will not disclose your information. Please feel free to fill it out.

3. As a participant, you may inquire about information and progress related to this study at any time. If you decide to withdraw, please inform us, and your data will not be included in the research results.

Finally, we sincerely thank you for taking the time out of your busy schedule to support our scientific research!

□I have been informed and agree that the collected data will be used for scientific research.

Informed Consent Signature:

Participation Date: year	month	day

| **Part I Basic Information** | |
| --- | --- |
| 1. **Your gender:** | a. Male b. Female |
| 2. **Your age: _____ years old. (Please set integer validation in Questionnaire Star)** | |
| 3. **Your place of residence:** | a. Rural b. Urban |
| 4. **Your educational level:** | a. Junior high school or below b. Senior high school / Vocational high school c. Junior college d. Bachelor’s degree e. Master’s degree or above |
| 5. **Your household’s per capita monthly income: _____ yuan** | a.<2000  b.2000-5000  c.5000-10000  d.10000-20000  e.>20000  F. Prefer not to disclose |
| 6. **Your marital status:** | a. Unmarried b. Married c. Other |
| 7. **Is this your first time wearing an orthodontic appliance?** | a. Yes, this is the first time b. No, I have been wearing one for a while |
| 8. **Your type of orthodontic treatment:** | a. Fixed appliance (bracket-type orthodontic appliance) b. Bracket-free clear aligners (invisible braces) c. Removable appliance (braces that can be taken off by oneself) |
| 9. **Duration of wearing your orthodontic appliance:** | a. Less than 3 months b. 3–6 months c. 6–12 months d. 1–2 years e. Over 2 years |
| 10. **Do you have any of the following dental or gum issues? (Multiple choices allowed)** | a. Gum bleeding b. Gum swelling c. Gum pain d. Loose teeth e. Dental caries (tooth decay) f. Tooth loss (missing teeth) g. None of the above |
| 11. **Do you have any family members or friends who have undergone orthodontic treatment?** | a. Yes b. No |
| 12. **What are your main sources of periodontal health information? (Multiple choices allowed)** | a. Doctor’s advice b. New media (WeChat official accounts / Xiaohongshu / Douyin, etc.) c. Traditional media (TV / radio / magazines / books, etc.) d. Communication with fellow patients e. Communication with family or friends |

| **Part 2 Knowledge of Periodontal Health** | | | |  |
| --- | --- | --- | --- | --- |
| **For the following statements, please indicate your level of understanding:** | | | | |
| **1. Periodontitis is caused by a variety of factors including poor oral hygiene, smoking, and genetic predisposition.** | a. very familiar | b. heard of it | c. not clear | |
| **2.** **Orthodontic treatment may increase the risk of periodontal problems such as gingival inflammation.** | a. very familiar | b. heard of it | c. not clear | |
| **3.** **Bleeding gums may be an early symptom of periodontal disease.** | a. very familiar | b. heard of it | c. not clear | |
| **4. Periodontal disease may lead to loose teeth or even tooth loss.** | a. very familiar | b. heard of it | c. not clear | |
| **5.** **Periodontal disease can be prevented through regular cleaning and proper care.** | a. very familiar | b. heard of it | c. not clear | |
| **6. Proper brushing techniques can effectively prevent periodontal diseases.** | a. very familiar | b. heard of it | c. not clear | |
| **7.** **Using dental floss helps clean interdental spaces and reduces gingivitis.** | a. very familiar | b. heard of it | c. not clear | |
| **8. Regular dental check-ups help with early detection of periodontal problems.** | a. very familiar | b. heard of it | c. not clear | |
| **9. Periodontal disease not only affects oral health but may also impact overall health.** | a. very familiar | b. heard of it | c. not clear | |

| **Part 3 Attitudes Toward Periodontal Health** | | | | | |
| --- | --- | --- | --- | --- | --- |
| 1. **I believe maintaining good periodontal health is important during orthodontic treatment.** | a. strongly agree | b. agree | c. neutral | d. disagree | e. strongly disagree |
| **2. I believe periodontal health affects the effectiveness of orthodontic treatment.** | a. strongly agree | b. agree | c. neutral | d. disagree | e. strongly disagree |
| **3. I believe patients should have regular periodontal check-ups after orthodontic treatment.** | a. strongly agree | b. agree | c. neutral | d. disagree | e. strongly disagree |
| **4. I believe orthodontic patients should receive professional guidance on periodontal health.** | a. strongly agree | b. agree | c. neutral | d. disagree | e. strongly disagree |
| **5. I am concerned about periodontal health issues occurring during orthodontic treatment.** | a. strongly agree | b. agree | c. neutral | d. disagree | e. strongly disagree |
| **6. I believe that good oral hygiene habits can prevent most periodontal problems.** | a. strongly agree | b. agree | c. neutral | d. disagree | e. strongly disagree |
| **7. I trust my dentist's professional competence regarding periodontal health.** | a. strongly agree | b. agree | c. neutral | d. disagree | e. strongly disagree |
| **8. I am willing to follow the dentist’s instructions for daily oral cleaning and care.** | a. strongly agree | b. agree | c. neutral | d. disagree | e. strongly disagree |
| **9. Orthodontic treatment has made maintaining my oral hygiene more difficult.** | a. strongly agree | b. agree | c. neutral | d. disagree | e. strongly disagree |
| **10. I am willing to learn more about periodontal health during orthodontic treatment.** | a. strongly agree | b. agree | c. neutral | d. disagree | e. strongly disagree |

| **Part 4 Practices Related to Periodontal Health**  Always: nearly 100% of the time; Often: about 70% or more; Sometimes: about 40–70%; Rarely: about 10–40%; Never: less than 10% or not at all | | | | | |
| --- | --- | --- | --- | --- | --- |
| 1. **I attend orthodontic follow-up appointments at the hospital as scheduled by my doctor.** | a. always | b. often | c. sometimes | d. rarely | e. never |
| 2. **I regularly visit the periodontal clinic for professional periodontal cleaning.** | a. always | b. often | c. sometimes | d. rarely | e. never |
| 3. **During orthodontic treatment, I follow the doctor’s advice for daily oral care.** | a. always | b. often | c. sometimes | d. rarely | e. never |
| 4. **I follow the doctor's instructions regarding daily cleaning of orthodontic appliances.** | a. always | b. often | c. sometimes | d. rarely | e. never |
| 5. **In daily life, I pay attention to the following:** |  |  |  |  |  |
| Using the correct brushing technique | a. always | b. often | c. sometimes | d. rarely | e. never |
| Brushing my teeth at least twice a day | a. always | b. often | c. sometimes | d. rarely | e. never |
| Using dental floss or other tools to clean between teeth | a. always | b. often | c. sometimes | d. rarely | e. never |
| Using mouthwash to maintain oral hygiene | a. always | b. often | c. sometimes | d. rarely | e. never |
| 6. **When I experience oral discomfort, I promptly go to the hospital and seek advice from healthcare professionals.** | a. always | b. often | c. sometimes | d. rarely | e. never |
