# Supplementary material for: Orthodontic patients’ knowledge, attitude, and practice regarding periodontal health
Source: BMC Oral Health. 2026 Jun 3;26:1569. doi: 10.1186/s12903-026-08750-4 (PMC13504998; doi:10.1186/s12903-026-08750-4)
Supplement: Supplementary file 2 [file 12903_2026_8750_MOESM3_ESM.docx]

**Supplementary Figure legend**

**Figure S1. (A) Self-reported periodontal symptoms among orthodontic patients. (B) Sources of information regarding orthodontic and periodontal care.**

**Supplementary table 1. Correlation analysis**

|  | Knowledge | Attitude | Practice |
| --- | --- | --- | --- |
| Knowledge | 1 |  |  |
| Attitude | 0.864 (0.854,0.874) *** | 1 |  |
| Practice | 0.873 (0.863,0.882) *** | 0.872 (0.863,0.881) *** | 1 |

Note: Correlation analyses were conducted using the total study population (N = 2,638).

Note on Confidence Interval Calculation for Table S1: All confidence intervals for Pearson correlation coefficients (r) in Table S1 were calculated using Fisher's z-transformation. Specifically, the correlation coefficient r was transformed to z’, the standard error was computed as 1/√(N-3) and the 95% CI was constructed on the z’ scale before being back-transformed to the r scale. This ensures accurate interval estimation irrespective of the sample size shared across comparisons.

*** P < 0.001.

**Supplementary table 2. SEM fit indicators**

| **Indicators** | **Reference** | **Results** |
| --- | --- | --- |
| RMSEA | <0.080 | 0.018 |
| SRMR | <0.080 | 0.014 |
| TLI | >0.800 | 0.990 |
| CFI | >0.800 | 0.989 |
